# Clinical Comparison of Three Blood-Derived Eye Drop Formulations for Moderate-to-Severe Dry Eye Disease: A Prospective Comparative Study

**DOI:** 10.7759/cureus.115216

**Published:** 2026-08-26

**Authors:** Konstantinos AA Douglas, Panagiotis Mallis, Vivian Paraskevi Douglas, Efstathios Michalopoulos, Konstantinos Armyros, Vasiliki Gkioka, Dimitrios Papaconstantinou, George Kymionis, Catherine Stavropoulos Giokas, Konstantinos Droutsas

**Affiliations:** 1 1st Department of Ophthalmology, General Hospital of Athens “G. Gennimatas”, National and Kapodistrian University of Athens, Athens, GRC; 2 Histocompatibility and Immunogenetics Laboratory, Hellenic Cord Blood Bank, Biomedical Research Foundation of the Academy of Athens, Athens, GRC; 3 Department of Ophthalmology, Beth Israel Deaconess Medical Center, Harvard Medical School, Boston, USA; 4 Blood Donation Unit, General Hospital of Athens “G. Gennimatas”, Athens, GRC; 5 Health and Social Care Management, University of West Attica, Athens, GRC

**Keywords:** autologous platelet lysate, autologous serum, biologic eye drops, cord blood platelet lysate, dry eye disease

## Abstract

Introduction

Dry eye disease (DED) is a multifactorial disorder of the ocular surface characterized by tear film instability, ocular discomfort, inflammation, and visual disturbance. Blood-derived eye drops have emerged as biologic therapeutic options for patients with moderate-to-severe DED because they contain growth factors and bioactive molecules that promote epithelial repair and restore ocular surface homeostasis. This study compared the short-term clinical performance and safety of autologous serum (AS), autologous platelet lysate (A-PL), and cord blood platelet lysate (CB-PL) eye drops.

Methods

This prospective randomized comparative study included 80 patients (160 eyes) with moderate-to-severe DED. Participants received AS (n = 20), A-PL (n = 20), or CB-PL (n = 40) for four weeks. Clinical evaluation included tear break-up time (TBUT), Oxford Grading Scale (OXFORD), Ocular Surface Disease Index (OSDI), Schirmer test, best-corrected visual acuity (VA), and intraocular pressure (IOP). Principal component analysis (PCA) was performed as an exploratory post hoc analysis to evaluate multidimensional treatment-response patterns.

Results

All three biologic formulations significantly improved TBUT, OXFORD score, and OSDI after four weeks of treatment. CB-PL demonstrated the largest median improvements across these clinical parameters, whereas A-PL showed intermediate improvement and AS produced clinically meaningful but comparatively smaller changes. No significant differences were observed among treatment groups for VA, Schirmer test, or IOP. No treatment-related adverse events, withdrawals, or protocol deviations occurred during follow-up.

Conclusion

All three blood-derived eye drop formulations improved both objective and subjective measures of DED after four weeks of treatment. CB-PL demonstrated the greatest magnitude of short-term clinical improvement in this cohort. These findings support the potential of platelet lysate-based therapies for patients with moderate-to-severe DED, although larger randomized studies with longer follow-up are needed to confirm these observations.

## Introduction

Dry eye disease (DED) is a multifactorial disorder of the ocular surface characterized by the loss of tear film homeostasis, accompanied by ocular symptoms in which tear film instability, hyperosmolarity, inflammation, ocular surface damage, and neurosensory abnormalities play major etiological roles [1,2]. It is one of the most prevalent ophthalmic disorders worldwide, affecting millions of individuals and representing a substantial socioeconomic burden because of its negative impact on visual function, productivity, and quality of life [3-8]. DED may result from aqueous tear deficiency, excessive tear evaporation, or a combination of both mechanisms, leading to chronic inflammation, epithelial damage, and progressive dysfunction of the lacrimal functional unit [2,4,9]. The prevalence of DED continues to increase owing to population aging, environmental factors, extensive digital device use, contact lens wear, refractive surgery, and systemic diseases, making it an important public health concern [3-10].

Conventional treatment of DED is based primarily on artificial tears, lubricating gels, anti-inflammatory medications, punctal occlusion, and environmental modifications [10-12]. Although these therapies improve lubrication and alleviate symptoms in many patients, they often fail to restore the biological components of the tear film that are essential for epithelial repair and maintenance of ocular surface homeostasis, particularly in moderate-to-severe disease [11-14]. Persistent epithelial damage and chronic inflammation may therefore continue despite conventional treatment, highlighting the need for biologically active therapeutic approaches capable of promoting tissue regeneration in addition to symptomatic relief [12-14].

Blood-derived eye drops have emerged as an important regenerative treatment for ocular surface disease because their biochemical composition closely resembles that of natural tears. These biologic formulations contain numerous bioactive molecules, including epidermal growth factor, transforming growth factor-β, platelet-derived growth factor, nerve growth factor, insulin-like growth factor-1, fibronectin, vitamins, cytokines, and other proteins that support epithelial proliferation, cellular differentiation, wound healing, neuromodulation, and maintenance of ocular surface integrity [12-19]. Unlike conventional lubricants, blood-derived eye drops actively contribute to tissue repair while simultaneously improving tear film stability and modulating ocular surface inflammation, making them particularly attractive for patients with persistent or severe DED [14-20].

Autologous serum (AS) remains the most widely used biologic tear substitute and has demonstrated favorable clinical outcomes in numerous studies involving severe DED and other ocular surface disorders [9,17-21]. Because its composition closely resembles that of natural tears, AS has become an established therapeutic option for promoting corneal epithelial healing and improving both objective clinical findings and patient-reported symptoms [9,18-21]. More recently, platelet-derived therapies have attracted increasing attention because platelet activation releases high concentrations of growth factors and regenerative mediators involved in tissue repair. Autologous platelet lysate (A-PL) provides a concentrated source of these biologically active molecules while avoiding exposure to allogeneic products, whereas cord blood platelet lysate (CB-PL) represents an alternative biologic formulation characterized by an enriched regenerative profile and abundant growth factors that may further support ocular surface healing [9,19-23].

Although the use of blood-derived eye drops has increased considerably during the last decade, direct prospective comparisons among AS, A-PL, and CB-PL remain limited. Most published studies have evaluated individual biologic formulations or compared them with conventional lubricants, making it difficult to determine their relative clinical performance using standardized outcome measures [9,20-23]. Comparative clinical evidence is therefore needed to better define the role of each biologic formulation in the management of moderate-to-severe DED.

The aim of the present prospective comparative study was to evaluate the short-term clinical performance and safety of AS, A-PL, and CB-PL eye drops in patients with moderate-to-severe DED. Treatment response was assessed using objective ocular surface parameters, patient-reported symptom scores, and exploratory multivariate analysis to provide a comprehensive comparison of these three blood-derived therapeutic formulations.

## Materials and methods

Study design

This prospective randomized comparative study was conducted at the Ophthalmology Department of GNA "G. Gennimatas" Hospital, Athens, Greece, from 18/10/2023 until 31/12/2024. The study was designed to compare the short-term clinical performance and safety of three blood-derived eye drop formulations, AS, A-PL, and CB-PL, in patients with moderate-to-severe DED. The study protocol was approved before participant enrollment, and all procedures were performed in accordance with the Declaration of Helsinki.

Ethics approval

The study was approved by the Scientific Council and Legal Department of GNA "G. Gennimatas" Hospital (Approval No. 27171), and the date of IRB approval was 18/10/2023. Written informed consent was obtained from all participants before enrollment. This study was registered under ClinicalTrials.gov identifier NCT07551843.

Study population

A total of 80 patients (160 eyes) with moderate-to-severe DED were prospectively enrolled in this comparative study. Eligible participants were adults (≥18 years of age) with persistent signs and symptoms of moderate-to-severe DED despite conventional therapy. The diagnosis was established based on clinical history and comprehensive ophthalmic examination, including a fluorescein tear break-up time (TBUT) of <10 seconds, Schirmer I test without anesthesia of ≤10 mm/5 minutes, corneal fluorescein staining graded as Oxford Grade II or higher according to the Oxford Grading Scale (OXFORD), and an Ocular Surface Disease Index (OSDI) score of ≥23, consistent with moderate-to-severe disease [2-7]. All participants were required to be able to comply with the prescribed treatment regimen and scheduled follow-up visits and to provide written informed consent before enrollment.

Patients were excluded if they had active ocular infection or acute ocular inflammation unrelated to DED, ocular surface neoplasia, severe eyelid abnormalities interfering with ocular surface evaluation, recent ocular surgery, current participation in another investigational treatment study, pregnancy or breastfeeding, or any systemic or ocular condition that, in the investigators' judgment, could interfere with study participation or interpretation of the results.

Randomization

Eligible participants were randomly assigned to receive AS, A-PL, or CB-PL using a computer-generated random allocation sequence. Although balanced enrollment across treatment groups was initially planned, recruitment into the autologous treatment groups was slower because several otherwise eligible patients declined autologous blood collection or additional venipuncture required for preparation of AS or A-PL. Consequently, recruitment into the CB-PL group was extended to maximize study enrollment while preserving the predefined eligibility criteria, treatment protocol, and follow-up schedule.

Preparation of blood-derived eye drops

Three blood-derived ophthalmic formulations were prepared under standardized sterile conditions: AS, A-PL, and CB-PL. All products underwent routine microbiological testing before clinical use. AS and A-PL were prepared at GNA "G. Gennimatas" Hospital according to standardized institutional protocols, whereas CB-PL was manufactured under Good Manufacturing Practice (GMP) conditions by the Hellenic Cord Blood Bank (HCBB) [19]. Preparation methods are summarized below.

Autologous Peripheral Blood Platelet Lysate

A 50 mL volume of peripheral blood was collected in anticoagulated tubes and centrifuged at 900 g for 10 minutes to isolate platelet-rich plasma (PRP). PRP was diluted to 30% with sterile saline, frozen at -80°C for at least 60 minutes, and rapidly thawed at 4°C to induce platelet lysis [22]. The lysate was aliquoted and stored at -20°C for approximately 45 days. On the day of use, vials were thawed at 4°C. After production, the eye drops were placed in sterile 5 mL and 10 mL containers with a dropper tip, thus ensuring ease of administration to the patients’ eyes. Approximately 7.5 mL of PL was obtained per 50 mL of whole blood.

Screening included HIV I/II, hepatitis B virus (HBV), hepatitis C virus (HCV), hepatitis A virus (HAV), human T-cell lymphotropic virus types I and II (HTLV I/II), West Nile virus (WNV), *Treponema pallidum*, *Trypanosoma cruzi*, and cytomegalovirus (CMV) (IgM/IgG). In the presence of any of these conditions, patients were required to report them in the informed consent form for participation in the study. Sterility testing for aerobic and anaerobic bacteria and fungi was performed using the BacT/Alert system, following European Pharmacopoeia standards.

Autologous Serum

For AS, 50 mL of peripheral blood was centrifuged at 4000 g at +8°C for 15 minutes. A total of 3.5 mL of saline from preservative-free eye drops was replaced with 3.5 mL of AS. Screening matched that of the PL group. Products were stored for one week at +4°C and up to four weeks at -20°C.

Umbilical Cord Blood Platelet Lysate

CB-derived PL was produced based on the PL production process according to the already published protocol of the HCBB [23-27], using cord blood units from full-term pregnancies (38-40 weeks) that were unsuitable for transplantation according to the criteria of the World Accreditation Organization “FACT-NetCord,” but were donated for research with informed consent, which was obtained prior to delivery and was in accordance with the National Bioethics Committee and the Helsinki Declaration. PRP was isolated via double centrifugation, frozen at -80°C for at least 24 hours, and rapidly thawed at 37°C to release platelet-derived growth factors. The lysate was filtered through a 0.22 μm non-pyrogenic filter and aliquoted into 5-10 mL vials. Up to 10 mL of PL was obtained per cord blood unit. Suitability criteria included platelet count >15 × 10⁹/L, WBC <4 × 10⁹/L, RBC <0.1 × 10¹²/L, and hemolysis <0.8%. CB eye drops were stored at -20°C for up to 30 days without measurable loss of growth factor content. Infectious disease and sterility testing followed the same standards as for autologous products.

Clinical evaluation

All participants underwent identical ophthalmic examinations before treatment initiation and after four weeks of therapy. Clinical evaluation included TBUT, Schirmer test without anesthesia, corneal fluorescein staining graded using the Oxford Grading Scale (OXFORD), best-corrected visual acuity (VA), intraocular pressure (IOP), and completion of the OSDI questionnaire. All examinations were performed using standardized procedures by experienced ophthalmologists.

Participants were instructed to instill one to three drops into each eye four times daily for four weeks. No changes to concomitant ocular medications were permitted during the study period.

Outcome measures

The primary outcome was the change in TBUT between baseline and Week 4. Secondary outcomes included changes in OXFORD score, OSDI, Schirmer test, VA, IOP, treatment tolerability, and safety.

Safety assessment

Safety was assessed at every study visit by slit-lamp examination and documentation of ocular and systemic adverse events. Particular attention was given to ocular inflammation, infection, allergic reactions, epithelial toxicity, discomfort, and treatment intolerance. No participant discontinued treatment because of adverse events.

Treatment compliance

Treatment adherence was excellent throughout the study. All participants completed the prescribed four-week treatment period and attended the scheduled follow-up examination. No withdrawals, protocol deviations related to treatment administration, or losses to follow-up occurred.

Exploratory principal component analysis (PCA)

PCA was performed as an exploratory post hoc multivariate analysis to investigate overall treatment-response patterns across the evaluated clinical parameters.

Continuous variables were standardized by mean-centering and scaling before PCA. Principal components were calculated using the prcomp function in RStudio (Posit Software, PBC, Boston, MA, USA). Score plots, loading plots, scree plots, and biplots were generated to visualize treatment-response patterns and variable contributions. Because PCA was not prespecified in the original statistical analysis plan, its findings should be interpreted as exploratory and hypothesis-generating.

Statistical analysis

Statistical analyses were performed using GraphPad Prism version 10.0 (GraphPad Software, Boston, MA, USA) and R (R Foundation for Statistical Computing, Vienna, Austria). Continuous variables are presented as median and interquartile range (IQR) unless otherwise specified. Within-group comparisons between baseline and post-treatment measurements were performed using the Wilcoxon signed-rank test. Comparisons among treatment groups or response clusters were conducted using the Kruskal-Wallis test, followed by Dunn's multiple-comparison test when appropriate. Statistical significance was defined as p < 0.05.

## Results

Patient enrollment and baseline characteristics

A total of 80 patients (160 eyes) with moderate-to-severe DED completed the study and were included in the final analysis. Twenty participants received AS, 20 received A-PL, and 40 received CB-PL. No participant withdrew from the study or was lost to follow-up, and treatment adherence was excellent in all treatment groups.

Baseline demographic characteristics are summarized in Table 1. The three treatment groups were comparable with respect to age and sex distribution, and no statistically significant differences were identified before treatment initiation (all p>0.05), indicating satisfactory baseline comparability among groups.

**Table 1:** Demographic characteristics of the three cohorts Data are presented as N for the total number of participants, n (%) for female participants, and median (IQR) for age. Abbreviations: AS, Autologous Serum; CB-PL, Cord Blood Platelet Lysate; A-PL, Autologous Platelet Lysate; IQR, Interquartile Range; N, total number of participants; n, number of participants in the category

Treatment group	N	Female (%)	Age, median (IQR) (years)
AS	20	55.0	60.0 (52.0-65.25)
CB-PL	40	72.5	59.5 (50.0-66.25)
A-PL	20	50.0	63.5 (54.25-69.25)

Primary clinical outcomes

**Figure 1:**
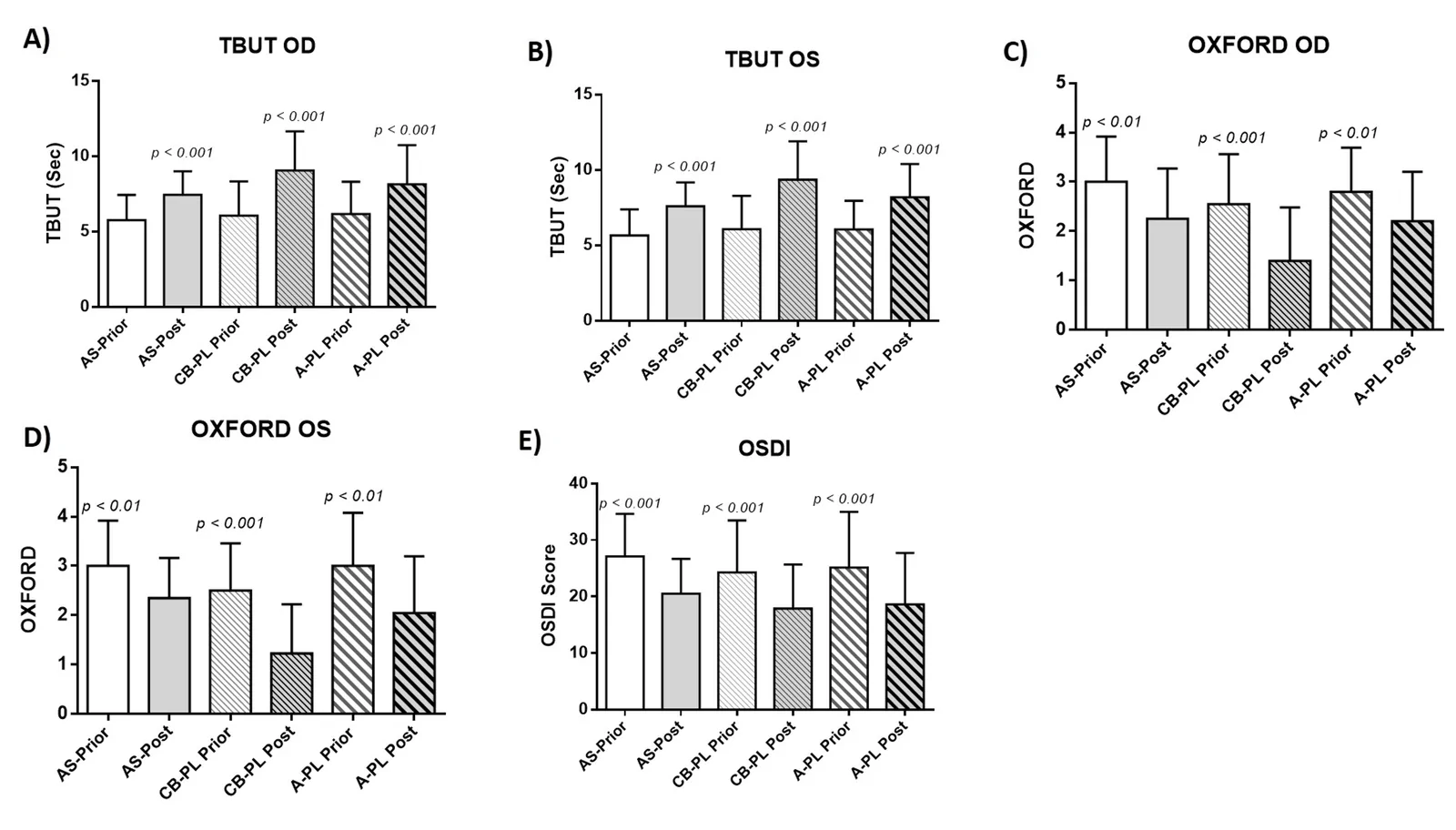
Clinical outcomes following four weeks of treatment with AS, A-PL, and CB-PL (A) TBUT in the right eye (OD). (B) TBUT in the left eye (OS). (C) Oxford Grading Scale (OXFORD) score in OD. (D) OXFORD score in OS. (E) OSDI score. Data were presented as mean ± standard deviation (SD). Statistical comparisons were performed using the Wilcoxon signed-rank test for within-group comparisons and the Kruskal-Wallis test with Dunn's multiple-comparison test for between-group comparisons. Statistical significance was defined as p < 0.05. Abbreviations: AS, Autologous Serum; CB-PL, Cord Blood Platelet Lysate; A-PL, Autologous Platelet Lysate; TBUT, Tear Break-Up Time; OSDI, Ocular Surface Disease Index

Changes in the principal clinical outcome measures following four weeks of treatment are presented in Figure 1.

All three biologic formulations produced statistically significant improvements in TBUT, OXFORD score, and OSDI [25] compared with baseline. Improvements were observed consistently in both eyes, demonstrating bilateral treatment effects across all study groups.

For TBUT, all treatment groups demonstrated prolonged tear film stability after therapy. The greatest median increase was observed in the CB-PL group, followed by A-PL, whereas AS also produced clinically meaningful improvement. Similar response patterns were observed in both the right (OD) and left (OS) eyes.

Corneal fluorescein staining, evaluated using the OXFORD grading scale, decreased significantly following treatment in all three groups, indicating improvement of ocular surface epithelial integrity. The largest reduction in staining scores was observed in patients receiving CB-PL, while A-PL demonstrated intermediate improvement and AS showed smaller but consistent reductions. Comparable findings between OD and OS suggested a uniform treatment response.

Patient-reported symptoms improved substantially after treatment. Median OSDI scores decreased in all treatment groups, indicating an overall reduction in patient-reported dry eye symptoms. The greatest reduction in OSDI was observed in the CB-PL group, followed by A-PL and AS.

Overall, platelet lysate-based formulations demonstrated larger median improvements across TBUT, OXFORD score, and OSDI than AS, although all three biologic therapies resulted in clinically meaningful improvements after four weeks of treatment.

Secondary clinical outcomes

The remaining clinical outcome measures are presented in Appendix 1.

VA remained stable throughout the study. No statistically significant differences were observed among treatment groups before treatment (OD: p = 0.96; OS: p = 0.89) or after treatment (OD: p = 0.92; OS: p = 0.98), indicating that none of the evaluated biologic formulations adversely affected visual function during the study period.

Similarly, IOP remained stable in all three treatment groups throughout follow-up, with no statistically significant differences observed before or after treatment (all p > 0.30).

Schirmer test values demonstrated modest increases following treatment in all groups. Numerically greater improvements were observed in participants treated with CB-PL; however, differences among treatment groups did not reach statistical significance for either eye (OD: p = 0.16; OS: p = 0.23).

These findings indicate that the principal treatment effects were observed in tear film stability, ocular surface integrity, and patient-reported symptoms, whereas VA, tear production, and IOP remained largely unchanged during the four-week follow-up period.

Exploratory PCA

**Figure 2:**
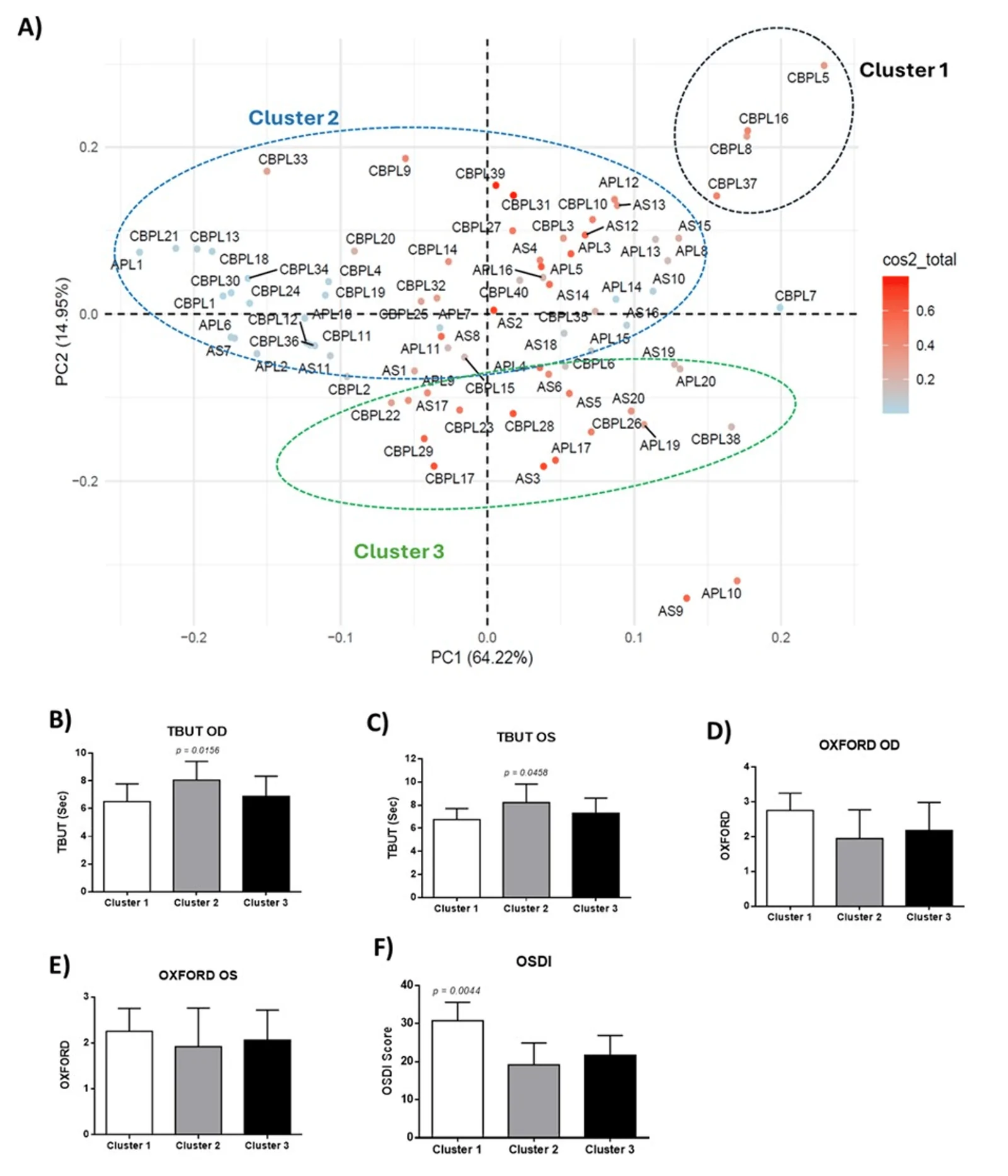
Exploratory PCA of treatment response and cluster-associated clinical profiles (A) PCA score plot showing participant-level observations and the three response clusters in the PC1-PC2 space (Cluster 1, n = 6 (9.5%); Cluster 2, n = 38 (60.3%); Cluster 3, n = 19 (30.2%)); PC1 and PC2 explain 64.22% and 11.45% of the variance, respectively. (B) Distribution of TBUT in the right eye (OD) across the three response clusters. (C) Distribution of TBUT in the left eye (OS) across the three response clusters. (D) Distribution of Oxford Grading Scale (OXFORD) score in the right eye (OD) across the three response clusters. (E) Distribution of OXFORD score in the left eye (OS) across the three response clusters. (F) Distribution of OSDI scores across the three response clusters. Data are presented as mean ± standard deviation (SD). Comparisons among response clusters were performed using the Kruskal-Wallis test followed by Dunn's multiple-comparison test. Statistical significance was defined as p < 0.05. Abbreviations: AS, Autologous Serum; CB-PL, Cord Blood Platelet Lysate; A-PL, Autologous Platelet Lysate; PC, Principal Component; TBUT, Tear Break-Up Time; OSDI, Ocular Surface Disease Index; OD, right eye; OS, left eye.

To further characterize multidimensional treatment-response patterns, an exploratory post hoc PCA was performed using all evaluated clinical parameters (Figure 2).

The first two principal components explained 75.7% of the total variance, indicating that most variability within the clinical dataset could be represented using a two-dimensional model.

Visual inspection of the PCA score plot demonstrated partial separation among treatment groups. Participants treated with CB-PL clustered predominantly within the region associated with greater improvements in TBUT, OXFORD score, and OSDI, whereas participants receiving AS occupied a distinct region corresponding to comparatively smaller overall clinical changes. The A-PL group demonstrated an intermediate distribution with partial overlap between the other two treatment groups.

The loading plot demonstrated that TBUT, OXFORD score, and OSDI contributed most strongly to the principal components, indicating that these variables accounted for the majority of treatment-related variability observed within the dataset.

Because PCA was performed as an exploratory post hoc analysis, these findings should be interpreted as hypothesis-generating and supportive of the observed clinical outcome patterns rather than as confirmatory evidence of comparative treatment effectiveness.

Cluster analysis

**Figure 3:**
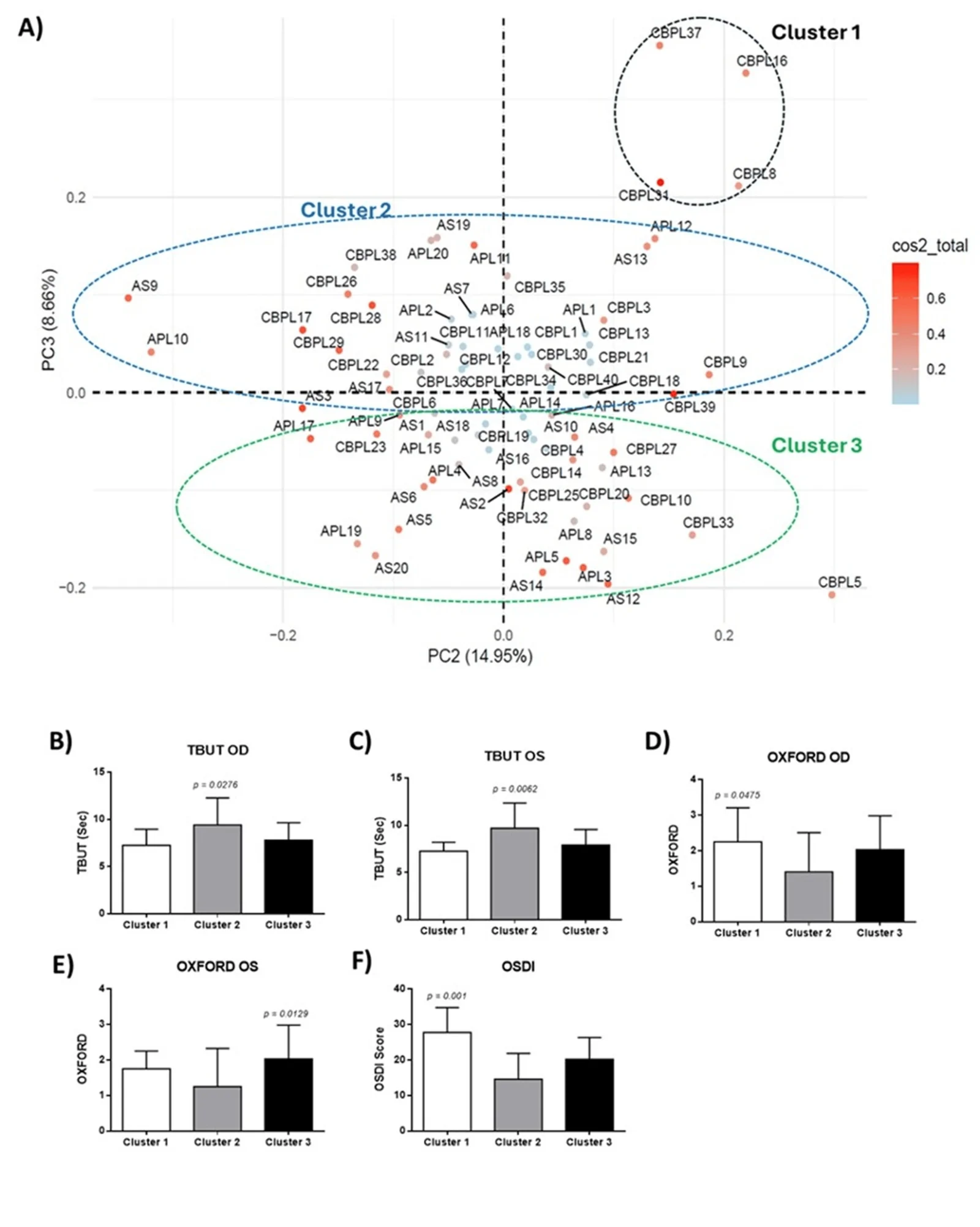
Hierarchical cluster analysis demonstrating three distinct treatment-response patterns (A) Clustered participant score plot in the PC2-PC3 space showing the three response clusters (Cluster 1, n = 6 (8.0%); Cluster 2, n = 34 (45.3%); Cluster 3, n = 35 (46.7%)). (B) Distribution of TBUT in the right eye (OD) across the three response clusters. (C) Distribution of TBUT in the left eye (OS) across the three response clusters. (D) Distribution of Oxford Grading Scale (OXFORD) score in the right eye (OD) across the three response clusters. (E) Distribution of OXFORD score in the left eye (OS) across the three response clusters. (F) Distribution of OSDI scores across the three response clusters. Data were presented as mean ± standard deviation (SD). Comparisons among response clusters were performed using the Kruskal-Wallis test followed by Dunn's multiple-comparison test. Statistical significance was defined as p < 0.05. Abbreviations: AS, Autologous Serum; CB-PL, Cord Blood Platelet Lysate; A-PL, Autologous Platelet Lysate; PC, Principal Component; TBUT, Tear Break-Up Time; OSDI, Ocular Surface Disease Index; OD, right eye; OS, left eye.

Hierarchical clustering identified three distinct response patterns among study participants (Figure 3).

Cluster 2 demonstrated the largest improvements in TBUT, OXFORD score, and OSDI, whereas Clusters 1 and 3 exhibited comparatively smaller overall clinical changes. Significant differences between Cluster 2 and the remaining clusters were observed across multiple ocular surface parameters (all p < 0.05), demonstrating heterogeneity in treatment response within the study population.

Despite these differences, measurable clinical improvement was observed across all three response clusters, indicating that each biologic formulation provided therapeutic benefit, although the magnitude of response varied among participants.

Safety

All three blood-derived eye drop formulations were well tolerated throughout the study period. No serious ocular or systemic adverse events were reported.

No participant discontinued treatment, and no withdrawals or losses to follow-up occurred. Furthermore, no clinically significant ocular inflammation, allergic reactions, epithelial toxicity, infectious complications, or treatment-related adverse events were observed during the four-week follow-up period.

These findings support the favorable short-term safety profile and tolerability of AS, A-PL, and CB-PL when prepared and administered under standardized conditions.

## Discussion

This prospective comparative study evaluated the short-term clinical performance and safety of three blood-derived eye drop formulations, AS, A-PL, and CB-PL, in patients with moderate-to-severe DED. All three treatments resulted in significant improvements in tear film stability, ocular surface staining, and patient-reported symptoms after four weeks of therapy. Among the evaluated formulations, CB-PL demonstrated the greatest magnitude of improvement in TBUT, OXFORD score, and OSDI, while A-PL showed intermediate improvement and AS also produced clinically meaningful therapeutic benefits.

These findings are consistent with the current understanding of DED as a chronic inflammatory disorder associated with tear film instability, epithelial damage, and impaired ocular surface homeostasis [1,2]. Conventional artificial tears primarily improve lubrication but do not replace the biological components necessary for epithelial regeneration and restoration of the ocular surface [3-7]. Consequently, biologic eye drops have become increasingly important in the management of moderate-to-severe DED because they contain growth factors, cytokines, extracellular matrix proteins, vitamins, and other bioactive molecules that actively support tissue repair while reducing ocular surface inflammation [7-19].

The clinical improvement observed with AS in the present study is consistent with previous reports demonstrating enhanced epithelial healing, improved tear film stability, and reduction of DED symptoms following AS therapy [8,21,22]. Because AS closely resembles the biochemical composition of natural tears, it has become an established treatment for severe ocular surface disease. Nevertheless, platelet-derived formulations have attracted increasing attention because platelet activation releases numerous regenerative mediators, including platelet-derived growth factor, epidermal growth factor, transforming growth factor-β, insulin-like growth factor, vascular endothelial growth factor, hepatocyte growth factor, and other molecules involved in epithelial proliferation, extracellular matrix remodeling, and tissue repair [13-19]. These biological characteristics may explain the greater clinical improvements observed in the platelet lysate groups in the present study.

Among the evaluated treatments, CB-PL demonstrated the largest improvements across the principal clinical outcome measures. Although the present study was not designed to investigate biological mechanisms, this observation is biologically plausible because cord blood-derived platelet lysate contains abundant growth factors and immunomodulatory mediators that may enhance epithelial regeneration while maintaining a favorable inflammatory profile [16-19]. Previous clinical studies have also reported encouraging outcomes with cord blood-derived biologic therapies in severe ocular surface disorders, including persistent epithelial defects, resistant corneal ulcers, and recalcitrant DED [21,22,27-29]. Although differences in study populations and treatment protocols preclude direct comparison, the present findings are consistent with the growing body of evidence supporting the regenerative potential of cord blood-derived ophthalmic therapies.

An exploratory PCA was performed to complement the conventional statistical analyses and evaluate multidimensional treatment-response patterns. The first two principal components explained 75.7% of the total variance, indicating that the principal clinical variables captured most of the variability within the dataset. The clustering pattern observed in the PCA score plots was consistent with the improvements demonstrated by conventional outcome measures, while hierarchical cluster analysis further illustrated the heterogeneity of treatment responses among participants. Because these analyses were performed post hoc, they should be considered exploratory and hypothesis-generating rather than confirmatory.

An important strength of this study is the prospective comparison of three clinically relevant blood-derived eye drop formulations using standardized objective examinations together with validated patient-reported outcome measures. Furthermore, all participants completed the study according to protocol, treatment adherence was excellent, and no treatment-related adverse events or withdrawals occurred, supporting the favorable short-term safety and tolerability of all three biologic formulations.

Several limitations should be acknowledged. The study included a relatively modest sample size, and treatment allocation was unequal because recruitment into the autologous treatment groups was limited by participant reluctance to undergo venipuncture for preparation of AS and A-PL. Although this affected group size, all enrolled participants completed treatment and follow-up according to the study protocol. In addition, the follow-up period was limited to four weeks, preventing assessment of long-term efficacy and durability of treatment response. Finally, PCA and hierarchical cluster analysis were performed as exploratory analyses and therefore require validation in larger prospective cohorts. Future multicenter randomized studies with longer follow-up, standardized manufacturing protocols, and biochemical characterization of blood-derived formulations are warranted to further define their role in the management of DED.

## Conclusions

Blood-derived eye drops represent an effective biologic treatment option for patients with moderate-to-severe DED. In this prospective comparative study, AS, A-PL, and CB-PL all produced significant improvements in tear film stability, ocular surface integrity, and patient-reported symptoms after four weeks of treatment while demonstrating excellent short-term safety and tolerability.

Among the evaluated formulations, CB-PL demonstrated the greatest magnitude of clinical improvement, whereas A-PL also showed favorable therapeutic performance compared with AS. Although these findings support the potential role of platelet lysate-based therapies in the management of moderate-to-severe DED, larger multicenter randomized clinical trials with longer follow-up and standardized biologic characterization are needed to confirm these observations and establish their long-term clinical effectiveness.

## Appendices

Appendix 1

**Table 2:** Pre-treatment and post-treatment comparison of clinical Data are presented as median (interquartile range (IQR)). Between-group comparisons for each parameter and time point were performed using the Kruskal-Wallis test. A two-sided p < 0.05 was considered statistically significant. Abbreviations: AS, Autologous Serum; CB-PL, Cord Blood Platelet Lysate; A-PL, Autologous Platelet Lysate; IQR, Interquartile Range; Pretx, Pre-treatment; PostTx, Post-treatment; VA, Visual Acuity; OD, Right Eye; OS, Left Eye; IOP, Intraocular Pressure

Parameter		AS median (IQR)	CB-PL median (IQR)	A-PL median (IQR)	Kruskal-Wallis p-value
VA Pretx	OD	0.05 (0-0.0925)	0.035 (0-0.10)	0.05 (0-0.0925)	0.96
	OS	0.045 (0.0175-0.085)	0.04 (0-0.10)	0.03 (0-0.09)	0.89
VA PostTx	OD	0.03 (0.00-0.08)	0.02 (0.00-0.10)	0.04 (0.00-0.08)	0.92
	OS	0.03 (0.00-0.08)	0.01 (0.00-0.10)	0.03 (0.00-0.08)	0.98
Schirmer Pretx	OD	6.65 (5.65-7.60)	7.55 (5.60-8.10)	6.60 (5.73-8.38)	0.76
	OS	6.80 (5.95-7.63)	7.15 (5.45-8.05)	7.25 (6.00-8.10)	0.79
Schirmer PostTx	OD	7.75 (6.48-8.63)	8.65 (7.40-10.30)	8.00 (6.25-9.03)	0.16
	OS	7.95 (6.93-8.53)	8.50 (7.50-10.20)	8.00 (6.98-9.03)	0.23
IOP Pretx	OD	15.0 (13-16.25)	13.0 (12-15)	14.0 (12-16)	0.30
	OS	14.0 (12.75-16)	13.5 (12-15.25)	14.0 (12-16)	0.69
IOP PostTx	OD	15.0 (12.75-15.25)	13.0 (12-15)	14.50 (12-15)	0.49
	OS	15.0 (13-15.25)	13.50 (12-15)	14.0 (12-16)	0.56
